# Editorial: Methodological Approaches for Quantitative Nursing Research

**DOI:** 10.2174/1874434601711010142

**Published:** 2017-10-31

**Authors:** Ileana Baldi, Dario Gregori

**Affiliations:** 1 Department of Cardiac, Thoracic and Vascular Sciences, University of Padova, Via Loredan 18, 35131 Padova, Italy; 2 Unit of Biostatistics, Epidemiology and Public Health, Department of Cardiac, Thoracic and Vascular Sciences, University of Padova, Italy

In recent decades the nursing discipline has begun to pay much greater attention to the need of an active participation by nurses in research [1]. Because nursing is an applied science that operates in a multidisciplinary perspective, an heterogeneous set of research approaches and language from many disciplines is used as normal machinery.

This volume probes in depth the methodological issues of a tool which is often used in nursing research to elicit the views of large groups of people: the questionnaire. Questionnaires can serve a variety of purposes within nursing research and allow researchers to quickly, effectively, and inexpensively evaluate programs, collect pilot data, and survey accessible populations, with the final aim to improve the nursing knowledge base. Rapidly increasing technology has fostered the use of electronic methodologies for developing and delivering questionnaires [2].

A practical guide for creating effective electronic questionnaires, adapted to the target population and able to collect relevant information is presented. Suggestions for designing questions and answers that are adequate measures of the topic of interest are offered. Findings from recent pilot surveys and evaluation projects which rely on questionnaires are presented.

Methods to account for interpersonal incomparability, related to how respondents may understand survey questions in different ways, alternative methods for agreement analysis and the network scale-up method for hard-to-count populations are just some of the statistical issues that are explored in detail in this monograph.

All the studies are intended to address issues relevant to the future development of the profession, by directing attention to sound methodological approaches for quantitative research.
